# Training of radiotherapy professionals: status, content, satisfaction and improvement suggestions in the Greater Region

**DOI:** 10.1186/s12909-022-03567-5

**Published:** 2022-06-22

**Authors:** Nadège Dubois, Anh Nguyet Diep, Alexandre Ghuysen, Joséfine Declaye, Anne-Françoise Donneau, Guillaume Vogin, Jochen Fleckenstein, Philippe Coucke, Selma Ben Mustapha

**Affiliations:** 1 Medical Simulation Center of Liège, Department of Public Health, Liège University, Liège, Belgium; 2 Department of Public Health, Biostatistics Unit, Liège University, Liège, Belgium; 3 UMR 7365 CNRS-UL IMOPA, Biopole, Vandoeuvre les Nancy, France; 4 Centre National de Radiothérapie du Luxembourg, Esch sur Alzette, Luxembourg; 5 Department of Radiotherapy and Radiation Oncology, Saarland University Medical Center, Homburg, Germany; 6 Department of Radiation Oncology, CHU of Liège, Liège, Belgium

**Keywords:** Radiotherapy, Training, Skills, Teaching methods

## Abstract

**Background:**

The initial training of Radiation Oncology professionals can vary widely across Europe. The aim of this study was to assess the status and content of the initial training programs currently implemented in the Greater Region: Lorraine (Nancy, France), Saarland (Homburg, Germany), Luxembourg, and Liège (Wallonia, Belgium).

**Methods:**

A survey was developed to investigate (1) the overall satisfaction, learning objectives, and teaching methods used during initial training programs and (2) the perceptions of the importance of key professional competencies as described by the CanMEDS (a framework that identifies and describes the abilities physicians require to effectively meet the health care needs of the people they serve). In addition, open-ended questions were used to elicit opinions on room for improvement. Participants (*N* = 38) were physicians (radiation oncologists (RO) seniors and residents) and radiation therapists (RTTs).

**Results:**

Only 21.1% of the respondents declared having acquired all the competencies required for their professional practice during their initial training. Heterogeneity in teaching methods was noted within professional programs but there is no difference between those from RO and RTT in the teaching of technical and relational skills. Relational skills were not addressed in a range of 39.5–57.9% of respondent’s curricula. More practical lessons were deemed necessary to improve radiotherapy (RT) training programs.

**Conclusions:**

Radiation oncology professionals expressed the need for more practical teaching, especially in the training of non-technical skills. Regarding the perceived importance of professional aptitudes, radiation oncology professionals highlighted medical and relational skills as the most important competencies.

**Supplementary Information:**

The online version contains supplementary material available at 10.1186/s12909-022-03567-5.

## Background

The burden of cancer is a global concern with 18.1 million of newly diagnosed cases a year and 9.6 million cancer-related deaths in 2018 [1]. The scientific community, supported by continuous technological advances, is constantly developing and improving therapies to address the issue. Currently, about 50% of cancer patients receive radiotherapy (RT) during their treatment process. Indeed, RT alone or in combination with other therapeutic modalities, improves the cure rate for 3.5 million people and provides palliative relief for an additional 3.5 million people [2, 3].

In 2008, the International Atomic Energy Agency (IAEA) drew attention on the heterogeneity of RT support worldwide and made recommendations on hospital infrastructures and staff training programs [4]. In Europe, an European society for Radiotherapy and Oncology (ESTRO) multidisciplinary survey assessed the organization, content, duration and cost of RT training programs, and found considerable variation among European countries despite attempts at standardization [5].

As a result, the ESTRO proposed timely updated core curricula available for each RT specialty based on theoretical knowledge and emphasizing on competency-based training programs [6, 7]. These curricula, based on the CanMEDS skills model that describes the seven roles carried out by all physicians [8], aim to define the minimum skills RT professionals may require to improve patients outcomes.

RT treatment involves multiple tasks and responsibilities in a complex interprofessional setting including not only radiation oncologists (RO) but also physicists and radiation therapists (RTT). Existing differences in their expectations and practices are challenging and question the need for professional skills training to work effectively as a team [9, 10].

In order to contribute to the joint-effort to standardize and update training programs in RT institutions in a European cross-border region (the Greater Region) [11], we investigated the status and content of the initial training programs currently implemented in universities and associated cancer treating centers in the Greater Region, as well as the satisfaction and improvement suggestions from a professional perspective. Based on the findings, we aimed to identify key elements for a relevant, standardized and updated training that echoes the expectations of the professional community as well as the quality requirements for daily practices.

## Methods

### Study design and procedures

An anonymous online survey using a protected document was sent through an open-source software (Google drive) to RO (including residents) and RTT (radiation therapists) working in RT departments and training institutions of the Interreg project in the Greater Region (EU-Interreg Va Greater Region Program N°043–1-01–125). Invitations to participate in the study were sent to the professional mailing lists of the institutions after approval by each RT head of department leading to a voluntary non-probability sampling method. The survey was kept open during 1 year from July 2019 to July 2020.

### Survey description

This survey was created by a radiation oncologist and senior author (SBM) and a pedagogical expert (ND) to get an overview of the initial training of RT professionals.

The survey first addressed the sociodemographic data of the respondents (4 items). The second part consisted in questions about the self-perceived satisfaction about the general organization (4 items; multiple choice), content and teaching materials used during the training (6 items about teaching materials and rating for relevance and adequacy). Content topics were divided into 3 categories: knowledge of basic and applied RT sciences, technical skills related to the workflow of cancer patients during the treatment process and finally non-technical skills.

Participants were also asked about the relevance of the topics taught in their training curriculum in view of their daily professional practice and the appropriateness of the teaching materials used by rating them using a 3-point Likert scale. Participants were then asked to rate (from 1 to 7) the CanMEDS professional skills in terms of their perceived importance in RT practice. Finally, open-ended questions were used to make suggestions for improving the training program. In total, the survey consisted of 18 items (see Additional file 1).

### Data analysis and statistical methods

Classical descriptive statistics were performed to describe the data. More specifically, frequencies and percentages were reported for qualitative variables, whereas median and interquartile ranges (Q25-75) were used to summarize quantitative variables due to non-normality. Furthermore, chi-square tests were applied to assess the association between the types of teaching methods used and the training programs of the participants. In case there were cells with expected count less than 5, Fisher’s exact test was employed. Once the omnibus chi-square test was significant, post-hoc examination using adjusted standardized residuals was performed to find out the significant associations. All results were considered to be significant at the 5% critical level (*p* < 0.05). Statistical analyses were carried out using R packages version 3.5.3.

For the analysis of open-ended questions, we used a combined approach for each individual question with a direct content analysis realized by one reviewer, followed by a summative content analysis. This allowed us to structure our process in identifying and creating coding categories. Concepts that did not belong to existing categories were grouped into an « others» category. Quantification focusing on frequency was then performed [12].

## Results

### Participants’ characteristics

A total of 40 questionnaires were obtained, of which two were removed because of incomplete data, yielding 38 valid questionnaires for analysis. The response rate was, therefore, 21.35% (number of professionals contacted = 178), with a completion rate of 95.00%. The majority of participants were between 31 and 40 years of age (50%, *n* = 19), and had initial training as Radiation Therapists (44.74%, *n* = 17) or as RO (seniors or residents) (42.11%, *n* = 16). Because RTTs may have multiple initial educational backgrounds, the survey included several response options: Nurse ± RT specialty, MIT ± RT specialty, and RTT.

Table 1 summarizes the socio-demographics data of the participants.

**Table 1:** Socio-demographic data of the participants (*N* = 38)

Categories	Number (%)
Gender	
Female	24 (63.16)
Male	14 (36.84)
Age (years)	
20–25	0 (0.00)
26–30	3 (7.89)
31–40	19 (50.00)
41–50	5 (13.16)
+ 50	11 (28.95)
Institution	
Sarre—Hombourg	4 (10.53)
Lorraine—Nancy	19 (50)
French high school	3 (7.89)
Liège	12 (31.58)
Initial training	
RO seniors and residents	16 (42.11)
RTT	22 (57.98)
Nurse	2 (5.26)
Nurse + RT specialty	2 (5.26)
MIT	0 (0)
MIT + RT specialty	1 (2.63)
RTT	17 (44.74)
Other	0 (0)

*RO* Radiation Oncologist, *RT* Radiotherapy, *MIT* Medical Imaging Technologist, *RTT* Radiation Therapists

### Self-perceived satisfaction of general organization and content

More than half of the participants (*n* = 22, 57.89%) felt that they had acquired an extended field of core competencies, and about 21.05% (*n* = 8) were confident that they possessed all the required ones as regards to their clinical daily practice (with significantly more RO). In contrast, 21.05% (*n* = 8) of respondents were not satisfied with the level of knowledge and competencies acquired. Most participants indicated that sufficient or too much time was spent on theoretical training (*n* = 31, 81.58%) and clinical work (*n* = 30, 78.94%). On the other hand, 52.63% (*n* = 20) advocated that the time spent on practical training was not sufficient. This lack of practical lessons was deemed stressful for 13.16% (*n* = 5) of respondents (see Additional file 2, Supplementary Tables 1–2). Only one third of the participants attended seminars abroad (*n* = 13, 34.21%), although 73.68% (*n* = 28) stated that seminars could be useful to their profession. Only 36.84% (*n* = 14) experienced simulation based medical education (SBME) during their training. Among professionals having received SBME, 85.70% reported a positive opinion (*n* = 12). Most of them pointed out the importance of SBME to fill the gap from theory to practice using scenarios that reflected reality, such as stressful or urgent cases (75%). Others reported a better understanding of anatomy (16.70%), and one participant also mentioned the interest of SBME to learn patient safety and security skills as well as error analysis (8.3%).

### Topics and teaching methods addressed in initial training

#### Theoretical knowledge

Regarding theoretical knowledge, 26.32% (*n* = 10) of the participants indicated that clinical oncology was not adequately covered in the curriculum. This lack was also observed for Radiation physics, general oncology, and radiotherapy techniques. Biological effects of radiation (median = 3 [2; 3]), general oncology (median = 3 [2; 3]), and radiotherapy techniques (median = 3 [2; 3]) were considered the most relevant topics to their professional practice. Regarding the adequacy of teaching support, more than half of participants considered that the teaching supports used to address biological effects of radiations, radiation physics, oncological pathology and radiotherapy techniques quite adequate. The majority indicated that the training they had received was fairly adequate (medians of ratings: 2) (see Additional file 2, Table 3).

Ex cathedra lecture was the most frequently mentioned teaching method (range 47.4–65.8%), followed by practical lessons (range 5.3–57.9%). About 25% of the participants added that e-learning was organized mainly in the teaching of radiation protection. SBME was only used in some cases, mainly in teaching clinical oncology (10.5%) and medical imaging (10.5%). Instructors tended to use more practical teaching for medical imaging. Other methods (range 10.5–28.9%) were also used, especially in teaching general and clinical oncology. Results are shown in Table 2.

**Table 2:** Teaching methods employed (n (%)) in the teaching of RT knowledge topics (*N* = 38)

Topics	Not addressed			Ex-cathedra			E-learning			Practical			SBME			Others			*p*-value
	**RO**	**RTT**	**Total**	**RO**	**RTT**	**Total**	**RO**	**RTT**	**Total**	**RO**	**RTT**	**Total**	**RO**	**RTT**	**Total**	**RO**	**RTT**	**Total**	
Radiation physics	0 (0)	3 (13.6)	3 (7.9)	14 (87.5)	10 (45.5)	24 (63.2)	2 (12.5)	0 (0)	2 (5.3)	2 (12.5)	1 (4.5)	3 (7.9)	1 (6.3)	0 (0)	1 (2.6)	0 (0)	9 (40.9)	9 (23.7)	< 0.001
Biological effects of radiation	0 (0)	2 (9.1)	2 (5.3)	14 (87.5)	11 (50)	25 (65.8)	2 (12.5)	0 (0)	2 (5.3)	2 (12.5)	0 (0)	2 (5.3)	1 (6.3)	0 (0)	1 (2.6)	0 (0)	9 (40.9)	9 (23.7)	< 0.001
Radiation protection	0 (0)	1 (4.5)	1 (2.6)	13 (81.3)	10 (45.5)	23 (60.5)	5 (31.3)	5 (22.7)	10 (26.3)	4 (25)	4 (18.2)	8 (21.1)	2 (12.5)	1 (4.5)	3 (7.9)	0 (0)	4 (18.2)	4 (10.5)	0.351
General oncology	0 (0)	1 (4.5)	1 (2.6)	13 (81.3)	10 (45.5)	23 (60.5)	3 (18.8)	0 (0)	3 (7.9)	5 (31.3)	0 (0)	5 (13.2)	2 (12.5)	1 (4.5)	3 (7.9)	0 (0)	11 (50)	11 (28.9)	< 0.001
Clinical oncology	0 (0)	0 (0)	0 (0)	13 (81.3)	11 (50)	24 (63.2)	3 (18.8)	0 (0)	3 (7.9)	8 (50)	5 (22.7)	13 (34.2)	2 (12.5)	2 (9.1)	4 (10.5)	0 (0)	10 (45.5)	10 (26.3)	0.002
Medical imaging	4 (25.0)	2 (9.1)	6 (15.8)	8 (50)	10 (45.5)	18 (47.4)	4 (25.0)	0 (0)	4 (10.5)	8 (50)	14 (63.6)	22 (57.9)	2 (12.5)	2 (9.1)	4 (10.5)	0 (0)	6 (27.3)	6 (15.8)	0.029
Radiotheraphy techniques	0 (0)	1 (4.5)	1 (2.6)	13 (81.3)	11 (50)	24 (63.2)	1 (6.3)	0 (0)	1 (2.6)	6 (37.5)	10 (45.5)	16 (42.1)	1 (6.3)	2 (9.1)	3 (7.9)	1 (6.3)	7 (31.8)	8 (21.1)	0.199

*Notes*: For each category, the percentage was calculated within each type of training; SBME: Simulation-based Medical Education

The association of teaching methods and initial training programs were investigated. Background training as Nurse, Nurse + RT specialty and MIT + RT specialty have been collapsed into the RTT group. Fisher’s exact tests revealed that the teaching methods employed were significantly different in the teaching of four RT knowledge topics. Accordingly, ex-cathedra (87.5%) was more observed in the RO training while other methods (40.9%) were more employed in the RTT training in the teaching of radiation physics (*p* < 0.001). Furthermore, other methods were also more prevalent in the teaching of biological effects of radiation (40.9%, *p* < 0.001), general oncology (50.0%, *p* < 0.001), and medical imaging (27.3%, *p* = 0.029) in the RTT training. In the RO training, on the other hand, practical lessons (31.3%) were more employed in the teaching of general oncology and e-learning in medical imaging (25.0%). Results are shown in Table 2.

#### Technical skills

Participants highlighted four items as being particularly relevant for their clinical practice: handling the initial outpatient consultation (median = 3, IQR: 2–3), the simulation/planning sessions (median = 3, IQR: 2–3), the short- and long-term patient follow-up (median = 3, IQR: 2–3), and the management of emergencies (median = 3, IQR: 2–3). All topics were perceived as quite relevant or highly relevant by the participants. In terms of appropriateness, a similar trend was observed. All participants reported that the skills related to the cancer patient workflow were adequately taught in the curriculum, with (median value of 2). However, 21.05% reported that management of emergencies was not adequately addressed; 18.42% reported that risk and incident management, contouring, dose prescription and dosimetry should have received more attention in the curriculum (see Additional file 2, Table 3).

Concerning technical skills, many participants reported that several topics were not covered in the curriculum. For more than half of the respondents, these included the initial outpatient consultation and quality management. Participants reported more hands-on teaching, particularly in simulation/planning session training (55.3%), contouring, dose prescription and dosimetry (55.3%). Ex-cathedra lectures remained the dominant approach (range: 13.2–47.4%). Interestingly, SBME, was mentioned by more participants. The topic that was strengthened by SBME was dose constraints for organs at risk (15.8%).

The teaching methods were found to be not different between the RO and RTT training in the teaching of technical skills in general. It was noted that the topic ‘undertake the initial outpatient consultation’ was largely not addressed in the RTT training (72.7%) and in terms of teaching methods, practical lessons were more observed in teaching this topic in the RO training (50.0%, *p* = 0.016). The result can be found in Table 3.

**Table 3:** Teaching materials employed in the teaching of technical skills (*N* = 38)

Topics	Not addressed			Ex-cathedra			E-learning			Practical			SBME			Others			*p*-value
	**RO**	**RTT**	**Total**	**RO**	**RTT**	**Total**	**RO**	**RTT**	**Total**	**RO**	**RTT**	**Total**	**RO**	**RTT**	**Total**	**RO**	**RTT**	**Total**	
Undertake the initial outpatient consultation	5 (31.3)	16 (72.7)	21 (55.3)	2 (12.5)	3 (13.6)	5 (13.2)	1 (6.3)	0 (0)	1 (2.6)	8 (50)	2 (9.1)	10 (26.3)	1 (6.3)	1 (4.5)	2 (5.3)	3 (18.8)	1 (4.5)	4 (10.5)	0.016
Treatment strategy according to the organ/area to be irradiated	1 (6.3)	4 (18.2)	5 (13.2)	9 (56.3)	9 (40.9)	18 (47.4)	3 (18.8)	0 (0)	3 (7.9)	8 (50)	5 (22.7)	13 (34.2)	3 (18.8)	2 (9.1)	5 (13.2)	3 (18.8)	7 (31.8)	10 (26.3)	0.229
Simulation session	5 (31.3)	2 (9.1)	7 (18.4)	6 (37.5)	8 (36.4)	14 (36.8)	1 (6.3)	0 (0)	1 (2.6)	7 (43.8)	14 (63.6)	21 (55.3)	2 (12.5)	3 (13.6)	5 (13.2)	2 (12.5)	5 (22.7)	7 (18.4)	0.436
Contouring, dose prescription, dosimetry	0 (0)	6 (27.3)	6 (15.8)	10 (62.5)	7 (31.8)	17 (44.7)	1 (6.3)	0 (0)	1 (2.6)	10 (62.5)	11 (50)	21 (55.3)	2 (12.5)	3 (13.6)	5 (13.2)	3 (18.8)	3 (13.6)	6 (15.8)	0.146
Organs at risk dose constraints	0 (0)	3 (13.6)	3 (7.9)	10 (62.5)	8 (36.4)	18 (47.4)	1 (6.3)	0 (0)	1 (2.6)	10 (62.5)	5 (22.7)	15 (39.5)	3 (18.8)	3 (13.6)	6 (15.8)	3 (18.8)	7 (31.8)	10 (26.3)	0.183
Short and long term follow-up of the patient	4 (25)	9 (40.9)	13 (34.2)	6 (37.5)	7 (31.8)	13 (34.2)	1 (6.3)	0 (0)	1 (2.6)	8 (50)	1 (4.5)	9 (23.7)	2 (12.5)	1 (4.5)	3 (7.9)	2 (12.5)	4 (18.2)	6 (15.8)	0.067
Risk and incident management	7 (43.8)	4 (18.2)	11 (28.9)	2 (12.5)	8 (36.4)	10 (26.3)	2 (12.5)	1 (4.5)	3 (7.9)	4 (25)	3 (13.6)	7 (18.4)	1 (6.3)	1 (4.5)	2 (5.3)	3 (18.8)	8 (36.4)	11 (28.9)	0.229
Quality management	7 (43.8)	13 (59.1)	20 (52.6)	4 (25)	5 (22.7)	9 (23.7)	1 (6.3)	0 (0)	1 (2.6)	4 (25)	0 (0)	4 (10.5)	1 (6.3)	0 (0)	1 (2.6)	4 (25)	4 (18.2)	8 (21.1)	0.134
Medical informatics	4 (25)	9 (40.9)	13 (34.2)	5 (31.3)	5 (22.7)	10 (26.3)	1 (6.3)	0 (0)	1 (2.6)	6 (37.5)	7 (31.8)	13 (34.2)	1 (6.3)	1 (4.5)	2 (5.3)	3 (18.8)	3 (13.6)	6 (15.8)	0.851
Management of emergency cases	2 (12.5)	9 (40.9)	11 (28.9)	8 (50)	4 (18.2)	12 (31.6)	1 (6.3)	0 (0)	1 (2.6)	6 (37.5)	3 (13.6)	9 (23.7)	2 (12.5)	2 (9.1)	4 (10.5)	2 (12.5)	5 (22.7)	7 (18.4)	0.089

*Notes*: For each category, the percentage was calculated within each type of training; SBME: Simulation-based Medical Education

#### Relational skills

All participants recognized the relevance of non-technical skills to their professional practice. Among these, teamwork (median = 3.0, IQR: 2–3) and interprofessional communication (median = 3.0, IQR: 2–3), were given the highest ranking. Teamwork (mean = 2.0, IQR: 2.0–2.75) was deemed highly adequate by the participants. For other non-technical skills, some respondents indicated that interprofessional communication (13.16%) and communication with patients and their relatives (13.16%) were not adequately addressed (see Additional file 2, Table 3).

The teaching of relational skills appeared to be less prevalent in the present sample and even absent from the curriculum of almost half of the participants. Teaching of non-technical skills was mostly done during practical (range 13.2–34.2%), SBME (range 5.3–15.8%) or ex-cathedra lessons (range 10.5–28.9%). The skill that was mostly taught by practical (34.2%) and SBME (15.8%) was communication with patients and their relatives. No significant differences in the teaching methods employed were observed in the RO and RTT programs as presented in Table 4.

**Table 4:** Teaching materials used to teach relational skills (*N* = 38)

Topic	Not addressed			Ex-cathedra			E-learning			Practical			SBME			Others			*p*-value
	**RO**	**RTT**	**Total**	**RO**	**RTT**	**Total**	**RO**	**RTT**	**Total**	**RO**	**RTT**	**Total**	**RO**	**RTT**	**Total**	**RO**	**RTT**	**Total**	
Communication with patients and their relatives	5 (31.3)	10 (45.5)	15 (39.5)	1 (6.3)	4 (18.2)	5 (13.2)	0 (0)	0 (0)	0 (0)	8 (50)	5 (22.7)	13 (34.2)	4 (25)	2 (9.1)	6 (15.8)	3 (18.8)	2 (9.1)	5 (13.2)	0.321
Patient's therapeutic education	6 (37.5)	16 (72.7)	22 (57.9)	4 (25)	2 (9.1)	6 (15.8)	1 (6.3)	0 (0)	1 (2.6)	5 (31.3)	1 (4.5)	6 (15.8)	1 (6.3)	1 (4.5)	2 (5.3)	3 (18.8)	3 (13.6)	6 (15.8)	0.079
Ethical standards	10 (62.5)	6 (27.3)	16 (42.1)	2 (12.5)	9 (40.9)	11 (28.9)	0 (0)	1 (4.5)	1 (2.6)	4 (25)	1 (4.5)	5 (13.2)	1 (6.3)	1 (4.5)	2 (5.3)	3 (18.8)	4 (18.2)	7 (18.4)	0.103
Interprofessional communication	10 (62.5)	10 (45.5)	20 (52.6)	1 (6.3)	3 (13.6)	4 (10.5)	0 (0)	1 (4.5)	1 (2.6)	4 (25)	3 (13.6)	7 (18.4)	1 (6.3)	2 (9.1)	3 (7.9)	3 (18.8)	4 (18.2)	7 (18.4)	0.920
Teamwork (collaboration, leadership, decision making)	11 (68.8)	11 (50)	22 (57.9)	0 (0)	4 (18.2)	4 (10.5)	0 (0)	1 (4.5)	1 (2.6)	3 (18.8)	3 (13.6)	6 (15.8)	1 (6.3)	2 (9.1)	3 (7.9)	2 (12.5)	4 (18.2)	6 (15.8)	0.504

*Notes*: For each category, the percentage was calculated within each type of training; SBME: Simulation-based Medical Education

Some professionals highlighted the satisfaction they obtained and the response to their needs, especially when less common teaching methods were used such as exchanges with clinical experts or clinical hands-on interventions. The most valued teaching tool for half of the respondents was practical, hands-on lessons (50.0%). Second in row, they preferred teaching that allows relational exchanges, such as work or discussions in small groups (29.4%), theoretical lessons (20.6%) and digital materials (17.6%). SBME and case studies were each reported by only one professional (total n for this question = 34).

### Professional competencies

When respondents were asked to rate the seven professional competencies defined in the CanMEDS model from 1 (least important) to 7 (most important) according to their own practice [8], 5 competencies were rated as important (median scores well above 4). These were professional, medical experts, followed by communicator, collaborator, and finally patient advocate. Leadership and scholarship were indicated as the least important competencies. The results are shown in Table 5.

**Table 5:** Median and interquartile ranges (IQR) of the ranking of professional competences (*N* = 38)

Professional competencies	Median [IQR]
Medical experts (theoretical and practical knowledge)	5.00 [3.00; 7.00]
Professional (ethical standard and excellence)	5.00 [2.00; 7.00]
Communicator (appropriate and effective communication)	4.00 [3.00; 6.00]
Collaborator (collaboration with other health professional)	4.00 [3.00; 6.00]
Patient advocate (supporter and advisor)	4.00 [3.00; 6.00]
Scholar (continuing education, teaching, and research)	3.00 [2.00; 4.00]
Leader (management of human and technical resources)	1.00 [1.00; 4.00]

Further analysis revealed almost no correlations between participants' age groups and gender and their ratings of the professional competencies, except in the area of scholarship. Accordingly, male participants (median = 4.0, IQR: 3.0–4.75) had a higher mean rank (*p* < 0.01) than female participants (median = 2.0, IQR: 2.0–3.25). Specific initial education and perceived level of knowledge acquisition were not significantly related to participants' professional competency rankings**.**

### Improvement strategies: qualitative analysis

At the end of the survey, participants were asked what courses could be added to their initial training (*n* = 38), the codification has led to the emergence of major themes covering teaching methods but also major crosscutting skills. Communication with patients and/or with other professionals was mentioned frequently (*n* = 10, 26.3%). They also expressed the need for more practice (*n* = 8, 21.1%) and more specific courses (*n* = 9, 23.7%) in the fields of dosimetry, medical oncology, and radiation therapy-specific software. Four professionals also mentioned a need for more teamwork training to improve inter- and intra-team collaboration and team management (10.5%). Other ways of improving training that were mentioned were medical simulation, ergonomics, hypnosis, time management and administrative matters.

## Discussion

The aim of this study was to provide an overview of the topics and teaching materials that have been used to teach radiation oncology in the Greater Region in recent decades. We also intended to explore the possibilities for improving radiotherapy education according to professional's opinions.

A recent study found that 16.7% of German young RO were not satisfied with their residency program [13]. Similarly, among Australian and New Zealand RO trainees, 7.5% reported that they were dissatisfied with their professional activities as trainees. Regarding their sense of self-efficacy, 54% were very satisfied with their feeling of being able to handle technical and non-technical aspects of RO, but 2.8% were not at all satisfied with this [14]. We found the same dichotomous pattern in our cohort, with 21.05% of participants reporting having acquired all the competencies required for their clinical practice on the one hand, and 21.05% of participants reporting that they had not acquired enough competencies on the other hand. Interestingly, one of the factors contributing to professional well-being has been shown to be related to workload or time management. Excessive workload and time pressure create stress and can eventually lead to burnout as reported by Leung et *al.*, which showed that 13% of trainees in RO suffered from one and/or the other [14]. In line with this, 13.16% of our population experience stress due to a lack of time.

A large proportion of participants, 39.47–57.89%, reported that relational skills were not addressed through their curriculum. Accordingly in the literature, only tumor-specific learning was mentioned in addition to basic science and training in RT technical skills without attention for non-technical skills training [5, 13, 15]. Therefore, the fact that more than a third of our surveyed population recommended to further emphasize non-technical skills in their education, such as communication and team training reflected the training needs from a professional perspective. In fact, the importance of these skills have been re-emphasized in the ESTRO core curricula and several small studies revealed that communicational workshop for professionals could improve not only self-efficacy but also patient satisfaction [16–18]. In view of these findings, it would be important to conduct surveys on this topic among a larger number of RO and RTT to confirm them. It would also be interesting to think about national or international initiatives offering specific soft skills courses for RO and RTT to fill this gap. This is important because we know that although the various tasks of RO and RTT are becoming more and more automated and artificial intelligence (AI) is in full expansion, the fact remains that patients like human contact with their caregivers and that soft skills like good communication and empathy are the basis of good patient care [19, 20].

Respondents rated all the teaching method mostly as quite adequate, or very adequate. Ex cathedra lectures and practical training lessons were the most commonly used to acquire knowledge and technical skills. Non-technical skills, despite not being covered very frequently, were mainly addressed through practical lessons, followed by ex-cathedra lectures and SBME. In the RT field, SBME appeared to be used to train various skills and procedural actions. A literature review by Rooney et al. found that more than half of the studies involved screen-based simulators and contouring exercises in particular. This review showed that SBME appeared to be more helpful than traditional teaching tools to learn specific radiation oncology skills [21]. In our cohort, we noticed that the teaching methods were found to be not different between the RO and RTT training in the teaching of technical skills and relational skills. In contrast, ex-cathedra, practical lessons and e-learning were more employed in the RO training for theoretical knowledge including: radiation physics, general oncology and medical imaging respectively. Indeed, it was demonstrated that in Europe, on average, 30% of medical student programs proposed e-learning for radiotherapy [22].

Web-based learning tools such as e-learning should not be underestimated as they enable the development of self-awareness and the improvement of radio-anatomical knowledge and treatment planning skills among RTT and RO trainees [23, 24]. It should however be noted that teaching methods, especially simulation modalities, should carefully be selected and fit the intended learning outcomes [25].

Regarding the seven professional competencies investigated, our study reveals that RT professionals overemphasize medical (medical expert and professional) and relational (communicator and collaborator) skills. The former is the most described and present in training programs, but the latter is perceived as important even if it is not well represented in the programs. Leadership comes last, although its importance in this radiation oncology was defined in a Delphi consensus study [26]. All competencies are valuable for each professional function and should be present in the curriculum of RT professionals, as reported by ESTRO and the updated curricula for RO and RTT [6, 7].

The small sample size of the study entails a limitation in the generalizability of the results, even though the findings are in accordance with previously published studies on the same topic. The limited number of participants may be due to the prior approval required from the heads of departments and also because the survey was launched during the COVID-19 pandemic.

## Conclusions

According to RT professionals working in the Greater Region, more practical lessons are needed to improve their training curriculum. Only one-fifth of the respondents declared having acquired all the competencies required for their professional practice during their initial training; and some of the professionals expressed the stress caused by the lack of time for practical lessons. Heterogeneity in teaching methods was noted within professional programs but there was no significant difference observed in the teaching methods employed to teach technical and relational skills in RO and RTT initial training programs. Furthermore, relational skills were not addressed in about half of the respondent’s curricula.

The findings are not groundbreaking, given that it has been recommended for many years that technical and non-technical skills must constitute an integral part of the RO and RTT curricula. Yet, the results obtained highlighted that certain prominent gaps between the initial training, the field expectations, and the learning needs from a professional perspective remain pertinent in this region. It is, therefore, essential to realize curriculum modifications, as prompted by this study, in the initial training. In addition, it is highly recommended to provide continuing education either on-site or from a distance targeting RO and RTT professionals to address these identified needs and ESTRO recommendations, e.g., using medical simulations teaching both technical and non-technical skills. This is because there has been a voiced demand as shown in the study, and it is only through these effort and initiatives that medical education will have an impact on the daily practices of health professionals.

## Supplementary Information

**Additional file 1. **Survey.

**Additional file 2. **Additional tables.

## Data Availability

The data generated or analysed during this study are available from the corresponding author on reasonable request.

## Abbreviations

ESTRO: European society for Radiotherapy and Oncology
IAEA: International Atomic Energy Agency
IQ: Interquartile ranges
RO: Radiation oncologist
RT: Radiotherapy
RTT: Radiation therapist
SBME: Simulation based medical education
TIM: Medical Imaging Technologist

## References

[1] Bray F, Ferlay J, Soerjomataram I, Siegel RL, Torre LA, Jemal A (2018). Global cancer statistics 2018: GLOBOCAN estimates of incidence and mortality worldwide for 36 cancers in 185 countries. CA Cancer J Clin.

[2] Jaffray DA, Knaul FM, Atun R, Adams C, Barton MB, Baumann M (2015). Global task force on radiotherapy for cancer control. Lancet Oncol.

[3] Barton MB, Jacob S, Shafiq J, Wong K, Thompson SR, Hanna TP (2014). Estimating the demand for radiotherapy from the evidence: a review of changes from 2003 to 2012. Radiother Oncol.

[4] International Atomic Energy Agency. Setting Up a Radiotherapy Programme: Clinical, Medical Physics, Radiation Protection and Safety Aspects. 2008. Available from: https://www-pub.iaea.org/MTCD/Publications/PDF/pub1296_web.pdf. [Cited 2018 Nov 16].

[5] Bibault JE, Franco P, Borst GR, Van Elmpt W, Thorwhart D, Schmid MP, et al. Learning radiation oncology in Europe: Results of the ESTRO multidisciplinary survey. Orig Res Artic. 2018; Available from: 10.1016/j.ctro.2018.02.001.. [Cited 2018 Dec 11]. 10.1016/j.ctro.2018.02.001 PMC5862689 29594252

[6] Eriksen JG, Beavis AW, Coffey MA, Leer JWH, Magrini SM, Benstead K (2012). The updated ESTRO core curricula 2011 for clinicians, medical physicists and RTTs in radiotherapy/radiation oncology. Radiother Oncol.

[7] Benstead K, Lara PC, Andreopoulos D, Bibault JE, Dix A, Eller YG (2019). Recommended ESTRO core curriculum for radiation oncology/radiotherapy 4th edition. Radiother Oncol.

[8] Frank JR, Samson DL (2015). Référentiels de compétences CanMEDS 2015 pour les médecins.

[9] Giddings A, Nica L, French J, Davis CA, Smoke M, Bolderston A (2018). Patterns of practice in Canadian radiation treatment centres: results of a national survey. J Med imaging Radiat Sci.

[10] Martens B, Veldman L, Singleton M, Fawcett S, Ali S (2018). Radiation therapists’ perceptions of advanced practice in Alberta. J Med imaging Radiat Sci.

[11] Vogin G, Fleckenstein J, Servotte JC, Nickers P, Ebersberger A, Mohammad F (2018). NHL-ChirEx: an interprofessional cross-border education initiative in the Greater Region with a focus on radiation morbidity and patient safety. Radiother Oncol.

[12] Hsieh HF, Shannon SE (2005). Three approaches to qualitative content analysis. Qual Health Res.

[13] Dietzel CT, Jablonska K, Niyazi M, Gauer T, Ebert N, Ostheimer C (2018). Quality of training in radiation oncology in Germany: where do we stand?: Results from a 2016/2017 survey performed by the working group “young DEGRO” of the German society of radiation oncology (DEGRO). Strahlentherapie und Onkol.

[14] Leung J, Lehman M (2017). Radiation oncology directors of training survey 2016: Perspectives and challenges. J Med Imaging Radiat Oncol.

[15] Nabavizadeh N, Burt LM, Mancini BR, Morris ZS, Walker AJ, Miller SM (2016). Results of the 2013–2015 Association of Residents in Radiation Oncology Survey of Chief Residents in the United States. Int J Radiat Oncol Biol Phys.

[16] Gibon A-S, Merckaert I, Liénard A, Libert Y, Delvaux N, Marchal S (2013). Learning methods in radiation oncology Is it possible to improve radiotherapy team members’ communication skills? A randomized study assessing the efficacy of a 38-h communication skills training program. Radiother Oncol.

[17] Merckaert I, Delevallez F, Gibon AS, Liénard A, Libert Y, Delvaux N (2015). Transfer of communication skills to the workplace: impact of a 38-hour communication skills training program designed for radiotherapy teams. J Clin Oncol.

[18] Van Beusekom MM, Cameron J, Bedi C, Banks E, Humphris G (2019). Communication skills training for the radiotherapy team to manage cancer patients’ emotional concerns: a systematic review. BMJ Open.

[19] Gaetz L (2020). Perspective from a patient partner. J Med imaging Radiat Sci.

[20] Boon IS, Lim JS, Yap MH, Au Yong TPT, Boon CS (2020). Artificial intelligence and soft skills in radiation oncology: data versus wisdom. J Med Imaging Radiat Sci.

[21] Rooney MK, Zhu F, Gillespie EF, Gunther JR, McKillip RP, Lineberry M (2018). Simulation as more than a treatment-planning tool: a systematic review of the literature on radiation oncology simulation-based medical education. Int J Radiat Oncol Biol Phys.

[22] Ben Mustapha S, Meijnders P, Jansen N, Lakosi F, Coucke P (2019). The status of radiation oncology (RO) teaching to medical students in Europe. Clin Transl Radiat Oncol.

[23] Dungey G, Gallagher P (2018). Radiation therapy students’ perceptions of a wiki. Clin Teach.

[24] Alfieri J, Portelance L, Souhami L, Steinert Y, McLeod P, Gallant F (2012). Development and impact evaluation of an e-learning radiation oncology module. Int J Radiat Oncol Biol Phys.

[25] Chiniara G, Cole G, Brisbin K, Huffman D, Cragg B, Lamacchia M (2013). Simulation in healthcare: a taxonomy and a conceptual framework for instructional design and media selection. Med Teach.

[26] Turner S, Seel M, Trotter T, Giuliani M, Benstead K, Eriksen JG (2017). Defining a Leader Role curriculum for radiation oncology: a global Delphi consensus study. Radiother Oncol.

