# Supplementary material for: Training of radiotherapy professionals: status, content, satisfaction and improvement suggestions in the Greater Region
Source: BMC Med Educ. 2022 Jun 22;22:485. doi: 10.1186/s12909-022-03567-5 (PMC9217112; doi:10.1186/s12909-022-03567-5)
Supplement: Supplementary file 1 — Additional file 1. Survey. [file 12909_2022_3567_MOESM1_ESM.docx]

**Additional file 1 - Survey**

**Assessment survey for expectations and needs concerning the training of radiotherapy professionals.**

1. In your curriculum, did you discuss these notions, under which pedagogical support(s)? (check for yes)

|  | Not addressed | Ex-cathedra | E-learning | Practical | SBME | Others |
| --- | --- | --- | --- | --- | --- | --- |
| Radiation physics | □ | □ | □ | □ | □ | □ |
| Biological effects of radiation | □ | □ | □ | □ | □ | □ |
| Radiation protection | □ | □ | □ | □ | □ | □ |
| General Oncology | □ | □ | □ | □ | □ | □ |
| Clinical oncology | □ | □ | □ | □ | □ | □ |
| Medical imaging | □ | □ | □ | □ | □ | □ |
| Radiotherapy techniques | □ | □ | □ | □ | □ | □ |

1. In comparison with the previous table, evaluate the relevance* of the pedagogical support used, as well as the adequacy* of the courses with your daily practice. (1: Bad to 3: Very good)

|  | Not relevant : 1 | Quite relevant : 2 | Very relevant:3 | Not adequate : 1 | Quite adequate: 2 | Very adequate:3 |
| --- | --- | --- | --- | --- | --- | --- |
| Radiation physics | □ | □ | □ | □ | □ | □ |
| Biological effects of radiation | □ | □ | □ | □ | □ | □ |
| Radiation protection | □ | □ | □ | □ | □ | □ |
| General Oncology | □ | □ | □ | □ | □ | □ |
| Clinical oncology | □ | □ | □ | □ | □ | □ |
| Medical imaging | □ | □ | □ | □ | □ | □ |
| Radiotherapy techniques | □ | □ | □ | □ | □ | □ |

1. In your curriculum, did you discuss these notions, under which pedagogical support(s)? (check for yes)

|  | Not addressed | Ex-cathedra | E-learning | Practical | SBME | Others |
| --- | --- | --- | --- | --- | --- | --- |
| Undertake the initial outpatient consultation | □ | □ | □ | □ | □ | □ |
| Treatment strategy according to the organ / area to be irradiated | □ | □ | □ | □ | □ | □ |
| Simulation/planning session | □ | □ | □ | □ | □ | □ |
| Contouring, dose prescription, dosimetry | □ | □ | □ | □ | □ | □ |
| Organs at risk constraints | □ | □ | □ | □ | □ | □ |
| Short and long term follow-up of the patient | □ | □ | □ | □ | □ | □ |
| Risk and Incident management | □ | □ | □ | □ | □ | □ |
| Quality management | □ | □ | □ | □ | □ | □ |
| Medical Informatics | □ | □ | □ | □ | □ | □ |
| Management of emergency cases | □ | □ | □ | □ | □ | □ |

1. In comparison with the previous table, evaluate the relevance of the pedagogical support used, as well as the adequacy of the courses with your daily practice. (1: Bad to 3: Very good)

|  | Not relevant : 1 | Quite relevant : 2 | Very relevant:3 | Not adequate : 1 | Quite adequate: 2 | Very adequate:3 |
| --- | --- | --- | --- | --- | --- | --- |
| Undertake the initial outpatient consultation | □ | □ | □ | □ | □ | □ |
| Treatment strategy according to the organ / area to be irradiated | □ | □ | □ | □ | □ | □ |
| Simulation session | □ | □ | □ | □ | □ | □ |
| Contouring, dose prescription, dosimetry | □ | □ | □ | □ | □ | □ |
| Organs at risk constraints, treatments | □ | □ | □ | □ | □ | □ |
| Short and long term follow-up of the patient | □ | □ | □ | □ | □ | □ |
| Risk and Incident management | □ | □ | □ | □ | □ | □ |
| Quality management | □ | □ | □ | □ | □ | □ |
| Medical Informatics | □ | □ | □ | □ | □ | □ |
| Management of emergency cases | □ | □ | □ | □ | □ | □ |

1. In your curriculum, did you discuss these notions, under which pedagogical support(s)? (check for yes)

|  | Not addressed | Ex-cathedra | E-learning | Practical | SBME | Others |
| --- | --- | --- | --- | --- | --- | --- |
| Communication with patients and their relatives | □ | □ | □ | □ | □ | □ |
| Patient therapeutic education | □ | □ | □ | □ | □ | □ |
| Ethical standards | □ | □ | □ | □ | □ | □ |
| Interprofessional communication | □ | □ | □ | □ | □ | □ |
| Teamwork (collaboration, leadership, decision making) | □ | □ | □ | □ | □ | □ |

1. In comparison with the previous table, evaluate the relevance of the pedagogical support used, as well as the adequacy of the courses with your daily practice. (1: Bad to 3: Very good)

|  | Not relevant : 1 | Quite relevant : 2 | Very relevant:3 | Not adequate : 1 | Quite adequate: 2 | Very adequate:3 |
| --- | --- | --- | --- | --- | --- | --- |
| Communication with patients and their relatives | □ | □ | □ | □ | □ | □ |
| Patient therapeutic education | □ | □ | □ | □ | □ | □ |
| Ethical standards | □ | □ | □ | □ | □ | □ |
| Interprofessional communication | □ | □ | □ | □ | □ | □ |
| Teamwork (collaboration, leadership, decision making) | □ | □ | □ | □ | □ | □ |

If other pedagogical support used, which ones? ........................................................

Comment on pedagogical support? ..................................................

1. During your training (assistantship/internship) in radiotherapy-oncology,

During your training as a radiation therapist,

What kind of educational support did you appreciate most? What benefits did you get from it (at educational level)? Justify

…………………………………………………….

1. What kind of educational support did you appreciate the least? Justify …………………………………………
2. Concerning the distribution of training time:

| Training | Stressful, not enough time | Not enough time | Enough time | Too much time |
| --- | --- | --- | --- | --- |
| Theoretical lessons | □ | □ | □ | □ |
| Practical lessons | □ | □ | □ | □ |
| Clinical work | □ | □ | □ | □ |

1. Have you attended any courses or seminars abroad?

|  | Yes | No |
| --- | --- | --- |
| Yes/No | □ | □ |
| If so, did it help you? | □ | □ |
| If no, do you think it would have been useful? | □ | □ |

1. Did you received courses using medical simulation (high fidelity manikin, role games, virtual reality,..)?

- Yes
- No

If you have received any medical simulation lessons, what benefits did you get from it (in terms of learning knowledge and clinical practice)?

…………………………………………………………

1. According to you, rank these 7 skills in order of importance in the practice of a radiation oncologist or a radiation therapist.(7: the most important, 1: the least important)

|  | 1 | 2 | 3 | 4 | 5 | 6 | 7 |
| --- | --- | --- | --- | --- | --- | --- | --- |
| Professional (ethical standards and excellence) | □ | □ | □ | □ | □ | □ | □ |
| Leader (management of human and technical resources) | □ | □ | □ | □ | □ | □ | □ |
| Collaborator (collaboration with other health professionals) | □ | □ | □ | □ | □ | □ | □ |
| Medical expert (theoretical and practical knowledge) | □ | □ | □ | □ | □ | □ | □ |
| Scholar (continuing education, teaching, research) | □ | □ | □ | □ | □ | □ | □ |
| Patient advocate (supporter and advisor) | □ | □ | □ | □ | □ | □ | □ |
| Communicator (appropriate and effective communication) | □ | □ | □ | □ | □ | □ | □ |

1. After completion of your assistantship / internship, of your training,

Do you think you have acquired all the knowledge and skills necessary to practice the profession of radiation oncologist or radiation therapist?

- Yes
- A large majority, but not all
- No

1. If you could add one (or more) course(s) to this training to improve it, which one(s) will it be? Justify

………………………………………………………..

1. Age

| 20-25 | 26-30 | 31-40 | 41-50 | +50 |
| --- | --- | --- | --- | --- |
| □ | □ | □ | □ | □ |

1. Gender

| Female | Male |
| --- | --- |
| □ | □ |

1. Institution

| Hombourg-Sarre | Lorraine-Nancy | French high school | Liège |
| --- | --- | --- | --- |
| □ | □ | □ | □ |

1. Initial training

- Medicine + Radiotherapy-Oncology
- Nurse without additional training
- Nurse + training in radiotherapy
- Medical imaging technologist without additional training
- Medical Imaging Technologist + Training in radiotherapy
- Manipulator in radiotherapy, radiology
- Others
