# Supplementary material for: Training of radiotherapy professionals: status, content, satisfaction and improvement suggestions in the Greater Region
Source: BMC Med Educ. 2022 Jun 22;22:485. doi: 10.1186/s12909-022-03567-5 (PMC9217112; doi:10.1186/s12909-022-03567-5)
Supplement: Supplementary file 2 — Additional file 2. Additional tables. [file 12909_2022_3567_MOESM2_ESM.docx]

**Additional file 2 (Additional TABLES)**

Additional table 1. The perception of time distribution in initial RT professional’s training (N=38)

| **Course type** | **Stressful not enough time (%)** | **Not enough time (%)** | **Enough time (%)** | **Too much time (%)** |
| --- | --- | --- | --- | --- |
| Theoretical training | 3 (7.89) | 4 (10.53) | 24 (63.16) | 7 (18.42) |
| Practical work lessons | 5 (13.16) | 15 (39.47) | 18 (47.37) | 0 (0.00) |
| Clinical work | 1 (2.63) | 7 (18.42) | 28 (73.68) | 2 (5.26) |

Supplementary table 2. The perception of acquired competencies during initial training (N=38)

| **Acquired competencies** | **Number (%)** | | |
| --- | --- | --- | --- |
|  | **RO (n=16)** | **RTT (n=22)** | **Total (N=38)** |
| Possessed all the required competencies | 6 (37.5)* | 2 (9.1) | 8 (21.1) |
| Acquired an extended panel of competence | 5 (31.3) | 17 (77.3)* | 22 (57.9) |
| Not satisfied with the level of knowledge  and competence acquired | 5 (31.3) | 3 (13.6) | 8 (21.1) |

*Fisher exact test showed that there were significant differences in the ratings of the acquired competencies (p=0.024). Accordingly, there were more participants reported having possessed all the required competencies in the RO group and more participants reporting acquired only an extended panel of competence in the RTT group than expected.

Additional table 3. The perceptions of relevance and adequacy of the learning content

| **Learning topics** | **Relevance** | | | | **Adequacy** | | | |
| --- | --- | --- | --- | --- | --- | --- | --- | --- |
| **RT knowledge** | **Not relevant** | **Quite relevant** | **Very relevant** | **Missing** | **Not adequate** | **Quite adequate** | **Very adequate** | **Missing** |
| Radiation physics | 1 (2.63) | 17 (44.74) | 17 (44.74) | 3 (7.89) | 5 (13.16) | 20 (52.63) | 9 (23.68) | 4 (10.53) |
| Biological effects of radiation | 0 (0.00) | 15 (39.47) | 21 (55.26) | 2 (5.26) | 3 (7.89) | 22 (57.89) | 9 (23.68) | 4 (10.53) |
| Radiation protection | 1 (2.63) | 19 (50) | 17 (44.74) | 1 (2.63) | 3 (7.89) | 21 (55.26) | 12 (31.58) | 2 (5.26) |
| General oncology | 1 (2.63) | 17 (44.74) | 20 (52.63) | 0 (0.00) | 4 (10.53) | 22 (57.89) | 11 (28.95) | 1 (2.63) |
| Clinical oncology | 2 (5.26) | 18 (47.37) | 18 (47.37) | 0 (0.00) | 10 (26.32) | 14 (36.84) | 13 (34.21) | 1 (2.63) |
| Medical imaging | 1 (2.63) | 13 (34.21) | 18 (47.37) | 6 (15.79) | 2 (5.26) | 17 (44.74) | 12 (31.58) | 7 (18.42) |
| Radiotheraphy techniques | 1 (2.63) | 15 (39.47) | 21 (55.26) | 1 (2.63) | 4 (10.53) | 18 (47.37) | 14 (36.84) | 2 (5.26) |
| **Technical skills** | **Not relevant** | **Quite relevant** | **Very relevant** | **Missing** | **Not adequate** | **Quite adequate** | **Very adequate** | **Missing** |
| Undertake the initial outpatient consultation | 3 (7.89) | 5 (13.16) | 10 (26.32) | 20 (52.63) | 4 (10.53) | 7 (18.42) | 7 (18.42) | 20 (52.63) |
| Treatment strategy according to the organ/area to be irradiated | 2 (5.26) | 13 (34.21) | 17 (44.74) | 6 (15.79) | 2 (5.26) | 18 (47.37) | 12 (31.58) | 6 (15.79) |
| Simulation/planning session | 2 (5.26) | 14 (36.84) | 16 (42.11) | 6 (15.79) | 1 (2.63) | 19 (50) | 11 (28.95) | 7 (18.42) |
| Contouring, dose prescription, dosimetry | 4 (10.53) | 14 (36.84) | 14 (36.84) | 6 (15.79) | 7 (18.42) | 17 (44.74) | 8 (21.05) | 6 (15.79) |
| Organs at risk constraints | 2 (5.26) | 17 (44.74) | 15 (39.47) | 4 (10.53) | 5 (13.16) | 17 (44.74) | 11 (28.95) | 5 (13.16) |
| Short and long term follow-up of the patient | 1 (2.63) | 10 (26.32) | 14 (36.84) | 13 (34.21) | 2 (5.26) | 13 (34.21) | 10 (26.32) | 13 (34.21) |
| Risk and incident management | 4 (10.53) | 11 (28.95) | 11 (28.95) | 12 (31.58) | 7 (18.42) | 11 (28.95) | 8 (21.05) | 12 (31.58) |
| Quality management | 3 (7.89) | 10 (26.32) | 7 (18.42) | 18 (47.37) | 5 (13.16) | 11 (28.95) | 4 (10.53) | 18 (47.37) |
| Medical informatics | 3 (7.89) | 12 (31.58) | 11 (28.95) | 12 (31.58) | 5 (13.16) | 15 (39.47) | 5 (13.16) | 13 (34.21) |
| Management of emergency cases | 1 (2.63) | 12 (31.58) | 14 (36.84) | 11 (28.95) | 8 (21.05) | 12 (31.58) | 7 (18.42) | 11 (28.95) |
| **Relational skills** | **Not relevant** | **Quite relevant** | **Very relevant** | **Missing** | **Not adequate** | **Quite adequate** | **Very adequate** | **Missing** |
| Communication with patients and their relatives | 1 (2.63) | 12 (31.58) | 10 (26.32) | 15 (39.47) | 5 (13.16) | 14 (36.84) | 4 (10.53) | 15 (39.47) |
| Patient therapeutic education | 3 (7.89) | 8 (21.05) | 5 (13.16) | 22 (57.89) | 4 (10.53) | 9 (23.68) | 2 (5.26) | 23 (60.53) |
| Ethical standards | 2 (5.26) | 14 (36.84) | 6 (15.79) | 16 (42.11) | 4 (10.53) | 11 (28.95) | 5 (13.16) | 18 (47.37) |
| Interprofessional communication | 2 (5.26) | 5 (13.16) | 11 (28.95) | 20 (52.63) | 5 (13.16) | 9 (23.68) | 3 (7.89) | 21 (55.26) |
| Teamwork (collaboration, leadership, decision making) | 1 (2.63) | 6 (15.79) | 11 (28.95) | 20 (52.63) | 3 (7.89) | 10 (26.32) | 5 (13.16) | 20 (52.63) |
